# Literature review analysis of aortitis induced by granulocyte-colony stimulating factor

**DOI:** 10.3389/fphar.2024.1487501

**Published:** 2024-12-18

**Authors:** Ting Zhao, Huanhuan Xu

**Affiliations:** Department of Clinical Pharmacy, Weifang People’s Hospital, Shandong Second Medical University, Weifang, China

**Keywords:** recombinant human granulocyte-colony stimulating factor, aortitis, adverse event, cancer, chemotherapy

## Abstract

**Background:**

Recombinant human granulocyte-colony stimulating factors (G-CSF)-induced aortitis is a rare but particularly serious adverse event, commonly seen in cancer patients undergoing chemotherapy. The aim of this article is to clarify the clinical characteristics of G-CSF- induced aortitis and provide effective references for clinical diagnosis and intervention.

**Methods:**

Case reports of adverse reactions of aortitis induced by G-CSF were collected from the relevant databases. The patients’ basic information and adverse reaction process were recorded and subjected to descriptive analysis.

**Results:**

A total of 72 patients were enrolled, including 14 males and 58 females, with a mean age of 61.83 ± 10.30 years. The G-CSF type with the highest frequency of occurrence of aortitis is pegfilgrastim. Apart from three healthy stem cell donors, G-CSF-induced aortitis was primarily found in patients with underlying malignancies, especially in patients with breast cancer. The most common anticancer drugs used at onset were docetaxel, cyclophosphamide, and doxorubicin. CT scan showed that aortitis most commonly occured in the aortic arch and its branches. Most patients had a good prognosis, but 3 cases developed complications. Importantly, G-CSF-induced aortitis was also found in 4 asymptomatic patients.

**Conclusion:**

This article found that G-CSF-induced aortitis not only occured in cancer patients undergoing chemotherapy as previously reported in literature, but also in healthy stem cell donors. Especially, asymptomatic patients with G-CSF-induced aortitis faced a greater risk of being missed by the attending physician.

## 1 Introduction

Recombinant human granulocyte-colony stimulating factors (G-CSF) is a member of the hematopoietic growth factor family that mobilizes and increases peripheral blood hematopoietic stem cells in both blood donors and cancer patients. G-CSF is primarily used to promote an increase in neutrophil count during bone marrow transplantation and prevent chemotherapy-induced febrile neutropenia (Link, 2022). The common adverse reactions of G-CSF include fever, back pain, headache, bone pain, and myalgia. Severe adverse events include acute lung injury, acute coronary syndrome, and acute aortitis (D'Souza et al., 2008). According to Japanese Adverse Drug Event Report database, aortitis occurring after G-CSF administration is considered an adverse reaction of G-CSF (Oshima et al., 2019). Aortitis induced-by G-CSF is extremely rare, with an incidence rate of 0.3%–0.74% in patients with malignant neoplasms (Sasaki et al., 2021; Takamatsu et al., 2022; Lee et al., 2020).

Aortitis associated with G-CSF administration primarily presents as a systemic symptoms, manifesting as fever and increased levels of c-reactive protein (CRP) (Lee et al., 2020). Due to the similarity of symptoms, it is highly likely to be misdiagnosed as an infection, thereby delaying the timely treatment of aortitis. CT is one of the crucial diagnostic tools for definitive diagnosis.

Based on the cases reported to date, there are four types of G-CSF associated with aortitis, including filgrastim, lenograstim, pegfilgrastim and lipegfilgrastim. Filgrastim and lenograstim, are short-acting G-CSF, which have a similar structure and biological activity to endogenous human G-CSF (Lee et al., 2020). Pegfilgrastim and lipegfilgrastim are long-acting agent of G-CSF (Lee et al., 2020). Most notably, pegfilgrastim and filgrastim account for the majority of reported cases of aortitis. Although adverse events of arteritis caused by G-CSF have been reported in previous studies (Ito et al., 2023; Ito et al., 2024; Hoshina and Takei, 2019), adverse events of arteritis have not been described in large-scale cohort studies.

In the present study, we attempted to explore distribution, occurrence and combination therapy characteristics of G-CSF-induced aortitis. To achieve this aim, we analyzed data from case reports of aortitis induced by G-CSF published in databases.

## 2 Materials and methods

### 2.1 Patients selection

PubMed, MEDLINE, Embase, Web of Science, Science Direct, China National Knowledge Internet, Wan fang, and Wei Pu databases were searched until 23 March 2024. The keywords “arteritis,” “aortitis,” “large vessel vasculitis,” “adverse events,” and “granulocyte colony-stimulating factor” were used as search terms to collect case reports concerning adverse events of aortitis induced by G-CSF. The inclusion criteria were (Link, 2022) reports published in open journals and (D'Souza et al., 2008) studies including the patients’ basic information, symptoms, and treatment of adverse aortitis events. The exclusion criteria were (Link, 2022) cases repeated in the databases, and (D'Souza et al., 2008) articles with incomplete patient information, therapeutic process and prognosis of adverse events of aortitis.

### 2.2 Methods

A literature review research design included 44 papers, was accepted to collect well-documented information (Table 1). A total of 73 cases with aortitis induced by G-CSF were collected, with one case excluded due to insufficient information. For all 72 cases, age, sex, nationality, primary disease, types of G-CSF, combined chemotherapy regimen, onset time of aortitis adverse events, symptoms, aortitis extension, clinical intervention, and outcome were recorded. The relevant data was analysed statistically.

**TABLE 1:** Detailed summary of all G-CSF-induced aortitis symptoms in patients from case report.

NO.	Country/Year	Age/Sex	G-CSF Type/Treatment duration/Indication	Diagnose	Time to onset (days)	Treatment	Outcome/Complication
1 (Darie et al., 2004)	France/2003	55/F	Filgrastim/5 days/Blood stem cells graft	Healthy stem cell donors	6	Steroid	Improvement within 2 weeks/NO
2 (Adiga et al., 2009)	USA/2009	54/M	Filgrastim/8 ds/Neutropenia	Squamous cell carcinoma of the lung	13	Discontinuing	Improvement within 6 days/NO
3 (Miller et al., 2016)	Israel/2016	52/M	Filgrastim/4 days/Blood stem cells graft	Healthy marrow donor	180	Steroid	Improvement rapid/Aneurysm
4 (Umeda et al., 2016)	Japan/2016	78/F	Filgrastim/5 years/neutropenia	Cyclic neutropenia	30	Steroid	Improvement rapid/NO
5 (Sato et al., 2017)	Japan/2017	67/F	Pegfilgrastim/8 days/Neutropenia	Advanced lung adenocarcinoma	1	Steroid	Improvement rapid/Aortic dissection
6 (Parodis et al., 2018)	Sweden/2018	70/F	Filgrastim/1day/neutropenia	Breast cancer (HER2+)	9	Steroid	Improvement rapid/NO
7 (Parodis et al., 2018)	Sweden/2018	60/F	Filgrastim/1day/Neutropenia	Breast cancer (HER2+)	11	Steroid	Improvement rapid/NO
8 (Lardieri et al., 2018)	Germany/2013	Unknown/M	Pegfilgrastim/1day/Neutropenia	B cell lymphoma	15	Not reported	Improvement/NO
9 (Lardieri et al., 2018)	Japan/2016	49/F	Filgrastim/1 day/Neutropenia	Uterine cancer	6	NSAID	Improvement in 1 month/NO
10 (Lardieri et al., 2018)	Japan/2016	72/F	Pegfilgrastim/1 day/Neutropenia	Uterine cancer	13	No treatment	Improvement in 1.5 months/NO
11 (Lardieri et al., 2018)	Japan/2016	76/F	Pegfilgrastim/1 day/Neutropenia	Breast cancer	7	No treatment	Improvement in 11 days/NO
12 (Lardieri et al., 2018)	Japan/2016	77/F	Filgrastim/6 days/Neutropenia	Ovarian cancer	7	No treatment	Improvement in 1 months/NO
13 (Lardieri et al., 2018)	Japan/2017	47/F	Lenograstim/“several times”/Neutropenia	Ovarian cancer	8	Steroid	Resolved in 3 months/NO
14 (Lardieri et al., 2018)	Japan/2017	61/F	Pegfilgrastim/1day/Neutropenia	Breast cancer	7	Steroid	Resolved in 26 days; switched to filgrastim/NO
15 (Lardieri et al., 2018)	Japan/2017	62/F	Pegfilgrastim/1 day/Neutropenia	B cell lymphoma	12	Steroid	Resolved in 18 days/NO
16 (Lardieri et al., 2018)	Japan/2017	65/F	Pegfilgrastim/1 day/Neutropenia	Breast cancer	9	Steroid	Resolved in 1 month/NO
17 (Lardieri et al., 2018)	Japan/2017	66/M	Pegfilgrastim/1 day/Neutropenia	Prostate cancer	8	No treatment	Resolved in 1 month/NO
18 (Lardieri et al., 2018)	Japan/2017	69/F	Pegfilgrastim/1day/Neutropenia	Esophageal cancer	11	No treatment	Resolved in 11 days; switched to filgrastim/NO
19 (Harada et al., 2020)	Japan/2020	52/F	Filgrastim/3days/Neutropenia	Ovarian cancer	14	Steroid	Improvement rapid/NO
20 (Nakamura et al., 2020)	Japan/2020	66/F	Pegfilgrastim/1day/Prophylaxis	Breast cancer	11	Loxoprofen	Resolved in within 3 weeks/NO
21 (Soto Castillo et al., 2020)	Spain/2020	68/F	Filgrastim/3days/Prophylaxis	Breast cancer	5	Steroid	Resolved in within 4 days/NO
22 (Sugai et al., 2024)	Japan/2020	55/F	Lenograstim/6 months, Pegfilgrastim/10 days/neutropenia	Ovarian cancer	10	Antibiotics	Improvement/NO
23 (Sugai et al., 2024)	Japan/2020	69/F	Lenograstim/4 months, Pegfilgrastim/15 ays/neutropenia	Endometrial cancer	15	No treatment	Improvement/NO
24 (Sugai et al., 2024)	Japan/2020	59/F	Filgrastim/8 days/Neutropenia	Endometrial cancer	8	Steroid	Improvement/NO
25 (Sugai et al., 2024)	Japan/2020	74/F	Filgrastim/2 months, pegfilgrastim/15 days/Neutropenia	Esophageal cancer	8	No treatment	Improvement/NO
26 (Mukai et al., 2020)	Finland/2017	40/F	Lipegfilgrastim/1day/Neutropenia	Breast carcinoma	10	Steroid	Improvement/NO
27 (Mukai et al., 2020)	Finland/2016	53/F	Pegfilgrastim/1day/Neutropenia	Breast carcinoma	1	Steroid	Improvement/NO
28 (Mukai et al., 2020)	Finland/2018	56/F	Lipegfilgrastim/1day/Neutropenia	Lobular breast carcinoma	8	Steroid	Improvement/NO
29 (Mukai et al., 2020)	Finland/2018	70/F	Lipegfilgrastim/1day/Neutropenia	Breast carcinoma	5	Steroid	Improvement/NO
30 (Mukai et al., 2020)	Finland/2018	62/F	Filgrastim/3days, pegfilgrastim/3days /Neutropenia	Breast carcinoma	62	Steroid	Improvement/NO
31 (Mukai et al., 2020)	Finland/2018	52/F	Filgrastim/4days/Neutropenia	Breast carcinoma	4	Steroid	Improvement/NO
32 (Shirai et al., 2020)	Japan/2019	66/F	Pegfilgrastim/1 day/prophylaxis	Breast cancer	10	Steroid	Improvement rapid/NO
33 (Yamamoto et al., 2021a)	Japan/2020	65/F	Pegfilgrastim/1 day/prophylaxis	Pancreatic cancer	8	No treatment	Resolved in 10 days/NO
34 (Yamamoto et al., 2021a)	Japan/2020	74/F	Pegfilgrastim/1 day/prophylaxis	Tongue cancer	8	No treatment	Resolved in 10 days/NO
35 (Yamamoto et al., 2021a)	Japan/2020	47/F	Pegfilgrastim/1 day/prophylaxis	Uterine cancer	10	No treatment	Resolved in 14 days/NO
36 (Yamamoto et al., 2021a)	Japan/2020	43/F	Pegfilgrastim/1 day/prophylaxis	Uterine cancer	7	No treatment	Resolved in 21 days/NO
37 (Yamamoto et al., 2021b)	Japan/2021	78/F	Filgrastim/5 days/prophylaxis	B cell lymphoma	5	Steroid	Resolved in 7 day/NO
38 (Fujiwara et al., 2021)	Japan/2021	49/F	Lenograstim/1 week/neutropenia	Pinealoma	7	Steroid	Resolved in within 2 week/NO
39 (Kametani et al., 2021)	Japan/2021	66/F	Pegfilgrastim/7 days/neutropenia	Transverse colon adenocarcinoma	2	Steroid	Resolved in within 8 days/NO
40 (Costa Silva et al., 2021)	Japan/2021	56/M	Pegfilgrastim/1 day/prophylaxis	Extraosseous mucinous chondrosarcoma	4	Steroid	Resolved in within 6 days/NO
41 (Seto et al., 2022)	Portugal/2021	56/F	Pegfilgrastim/1 day/neutropenia	Breast cancer	3	Steroid	Resolved in within 5 day/NO
42 (Asif et al., 2022)	Japan/2022	48/F	Pegfilgrastim/1 day/neutropenia	Breast cancer	1	Steroid	Resolved in within 15 day/NO
43 (Mizushima et al., 2022)	UK/2022	64/F	Pegfilgrastim/1 day/prophylaxis	Breast cancer (HER2+)	7	Steroid	Resolved in within 21 days/NO
44 (Arnould et al., 2023)	Japan/2022	77/M	Filgrastim/1 day/prophylaxis	Lung neuroendocrine tumor	8	Steroid	Improvement rapid/NO
45 (Seto et al., 2023)	France/2023	73/F	Filgrastim/4 days/neutropenia	Breast cancer	5	No treatment	Resolved in within 45 days/NO
46 (Ozaki et al., 2023)	Japan/2023	71/M	Pegfilgrastim/1 day/prophylaxis	Large-cell neuroendocrine carcinoma	5	No treatment	Improvement rapid/NO
47 (Uemura et al., 2023)	Japan/2023	66/M	Pegfilgrastim/1 day/neutropenia	Prostate cancer	6	Steroid	Resolved in within 3 days/NO
48 (Nishioka and Fujii, 2023)	Japan/2023	45/M	Filgrastim/1 day/blood stem cells graft	Healthy stem cell donors	8	Steroid	Resolved in within 10 days/NO
49 (Iida et al., 2023)	Japan/2023	50/M	Pegfilgrastim/1 day/neutropenia	Neuroblastoma	8	No treatment	Resolved in within 10 days/NO
50 (Masuda et al., 2023)	Japan/2023	80/M	Pegfilgrastim /4 days/neutropenia	Bladder cancer	6	Steroid	Resolved in within 3 days/NO
51 (Koyama et al., 2021)	Japan/2023	83/F	Pegfilgrastim/3 days/prophylaxis	diffuse large B-cell lymphoma	3	Celecoxib	Resolved in within 7 days/NO
52 (Lee et al., 2020)	Korea/2020	45/F	Pegfilgrastim/5 days/neutropenia	Breast cancer	12	Steroid	Resolved in within 1 week/NO
			Filgrastim/1 day/neutropenia		10		Resolved in within 2 weeks / NO
53 (Lee et al., 2020)	Korea/2020	66/F	Pegflgrastim/3 days/neutropenia	Breast cancer	13	Steroid	Resolved in within 1 week/NO
54 (Lee et al., 2020)	Korea/2020	49/F	Pegfilgrastim/1 day/neutropenia	Breast cancer	15	Steroid	Resolved in within 1 week/NO
55 (Lee et al., 2020)	Korea/2020	50/F	Pegfilgrastim/1 day/neutropenia	Breast cancer	12	Steroid	Resolved in within 1 week/NO
56 (Lee et al., 2020)	Korea/2020	59/F	Pegfilgrastim/1 day/neutropenia	Breast cancer	17	Steroid	Resolved in within 1 week/NO
57 (Lee et al., 2020)	Korea/2020	53/F	Pegfilgrastim/4 days/neutropenia	Breast cancer	14	Steroid	Resolved in within 1 week/NO
58 (Tane et al., 2024)	Japan/2021	43/F	Pegfilgrastim/1 day/prophylaxis	Breast cancer	5	Steroid	Resolved in within 18 days/NO
59 (Ito et al., 2023)	Japan/2023	73/M	Pegfilgrastim/1 day/prophylaxis	Small-cell lung cancer	12	NSAIDS	Resolved in within 16 days/NO
60 (Ito et al., 2024)	Japan/2024	67/F	Pegfilgrastim/1 day/prophylaxis	Breast cancer	7	No treatment	Resolved in within 19 days/NO
61 (Sasaki et al., 2019)	Japan/2018	61/F	Pegfilgrastim/1 day/neutropenia	Breast cancer	1	No treatment	Resolved in within 10 days/NO
62 (Sasaki et al., 2019)	Japan/2019	72/F	Pegfilgrastim/1 day/neutropenia	Breast cancer	5	No treatment	Resolved in within 14 days/NO
63 (Hoshina and Takei, 2019)	Japan/2024	55/M	Filgrastim/10 days/prophylaxis	Diffuse large B-cell lymphoma	8	Steroid Steroid	Improvement rapid/NO
			Lenograstim/9 days/prophylaxis		5		Resolved in within 30 days / NO
64 (Kawahara et al., 2020)	Japan/2019	69/M	Pegfilgrastim/1 day/prophylaxis	Diffuse large B-cell lymphoma	13	No treatment	Resolved in within 7 days/NO
65 (Kawahara et al., 2020)	Japan/2019	62/F	Pegfilgrastim/1 day/prophylaxis	Diffuse large B-cell lymphoma	11	No treatment Steroid	Resolved in within 18 days/NO
			Pegfilgrastim/1 day/prophylaxis		12		Resolved in within 14 days/NO
66 (Saito et al., 2021)	Japan/2020	71/F	Pegfilgrastim/1 day/prophylaxis	Ovarian cancer	11	No treatment No treatment	Improvement rapid/NO
			Pegfilgrastim/1 day/prophylaxis		11		Resolved in within 27 days / NO
67 (Nitta et al., 2021)	Japan/2021	71/F	Pegfilgrastim/1 day/prophylaxis	Intrahepatic cholangiocarcinoma	7	Steroid	Resolved in within 21 days/NO
68 (Jimbo et al., 2021)	Japan/2021	72/M	Pegfilgrastim/1 day/prophylaxis	Prostatic cancer	10	Steroid	Resolved in within 18 days/NO
69 (Singh et al., 2022)	Japan/2021	58/F	Pegfilgrastim/2 days/prophylaxis	Breast cancer	8	No treatment	Resolved in within 26 days/NO
70 (Takahashi et al., 2022)	USA/2022	58/F	Pegfilgrastim/1 days/prophylaxis	Duodenal adenocarcinoma	7	NASIDs	Resolved in within 12 days/NO
71 (Matsumoto et al., 2022)	Japan/2022	55/F	Pegfilgrastim/1 days/prophylaxis	Pancreatic cancer	7	Steroid	Resolved in within 2 days/NO
72 (Mukai et al., 2020)	Japan/2022	63/F	Pegfilgrastim/1 days/prophylaxis	Breast cancer	7	No treatment	Resolved in within 20 days/NO
73 (Shiraki et al., 2022)	Japan/2022	70/F	Pegfilgrastim/1 days/prophylaxis	Breast cancer	8	Steroid	Resolved in within 445 days/Aortic dissection

### 2.3 Statistical analysis

Baseline characteristics were described as numbers and percentages for all patients treated with G-CSF. Continuous variables were presented as mean ± standard deviation (M±SD). Categorical variables were presented as percentages. All statistical analysis was performed using Graphpad Prism version 8.0.2.

## 3 Results

### 3.1 Frequency and clinical information related to G-CSF-induced aortitis

**FIGURE 1:**
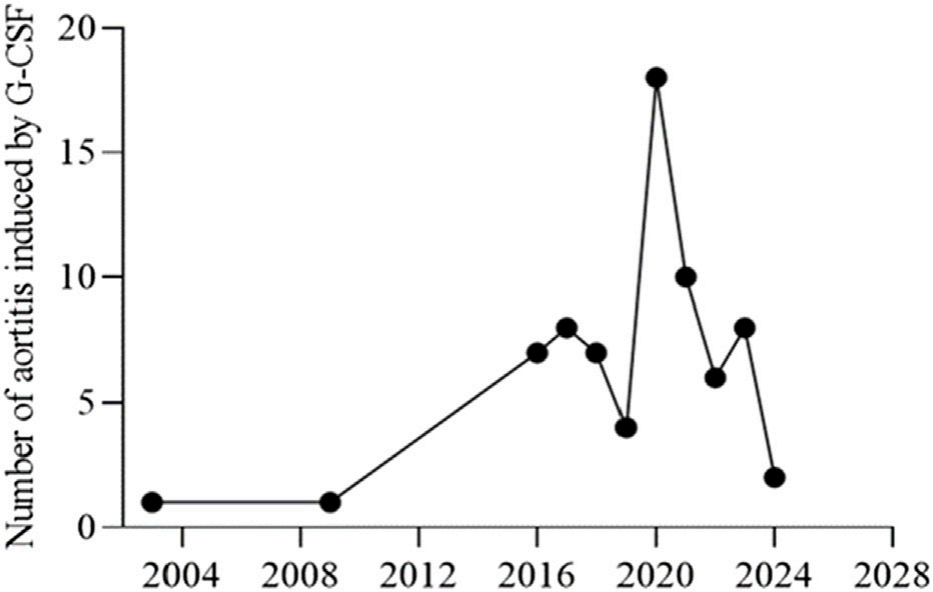
The annual trends in the number of G-CSF administrations.

**FIGURE 2:**
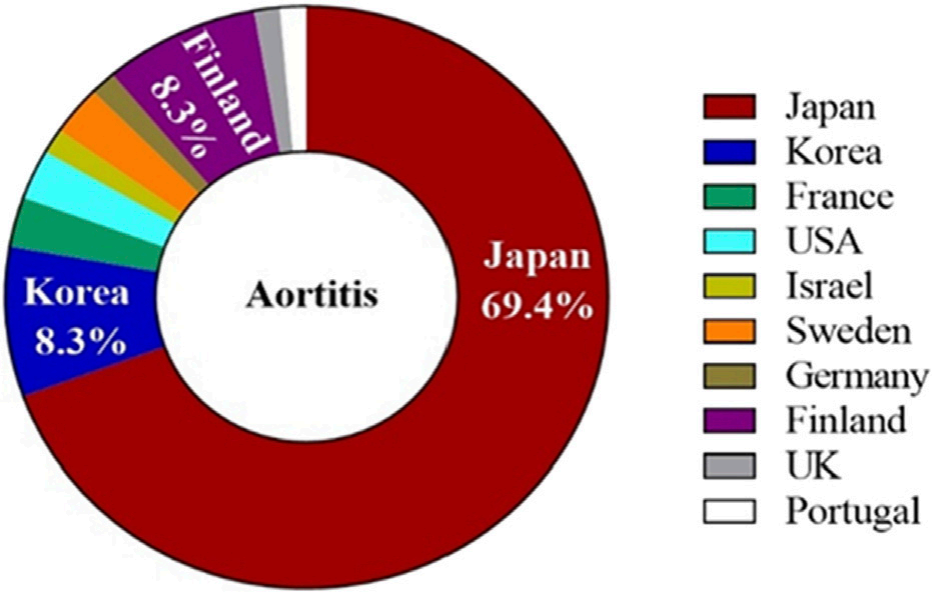
The distribution of G-CSF-induced aortitis in countries around the world.

The characteristics of the patients were presented in Table 2. There were 58 women and 14 men, with a mean age of 61.83 years ±10.30 (SD) (age range, 40–80 years) at the occurrence of adverse events related to aortitis. Patients with G-CSF-induced aortitis were predominantly female (male, 19.4%; female, 80.6%). The annual trends in the incidence of G-CSF-induced aortitis are shown in Figure 1, showing that the incidence of G-CSF-induced aortitis increased each year until 2020. Figure 2 shows the relationship between the incidence of G-CSF-induced aortitis and global distribution. The countries with the highest incidence of G-CSF-induced aortitis are Japan (50 cases, 69.4%), South Korea (6 cases, 8.3%) and Finland (6 cases, 8.3%). A total of 72 patients had a medical or prophylactic aim for filgrastim (n = 22), pegfilgrastim (n = 52), lenograstim (n = 5) or lipegfilgrastim (n = 3) during the identification period (Table 2). Of all, pegfilgrastim was characterized by the highest frequency (63.4%) of arteritis. Patients with G-CSF-induced aortitis of these four types were remarkably similar in terms of sex, age and time to onset. Patients who use filgrassim (CRP 26.06 ± 15.39 mg/dL), pegfililgrassim (CRP 24.81 ± 9.65 mg/dL), and lenograstim (CRP 10.45 ± 9.914 mg/dL) all presented varying degrees of elevated serum CRP levels (Table 3). Unfortunately, no CRP related data were provided for the four cases using lipegfilgrastim. In addition, Patients with G-CSF-induced aortitis of these four types were similar in clinical manifestations, such as fever, chest pain, abdominal pain, neck pain, back pain, earache, sore throat, headache and myalgia, etc. Meanwhile, the four types of G-CSF-induced aortitis also exhibited similarities in their common occurrence sites, all of which were prone to occur in the aortic arch, abdominal aorta, and thoracic aorta, etc.

**TABLE 2:** Baseline characteristics of Reported Patients.

Characteristic	Aortitis (n = 72)
Sex	
Female, n (%)	58 (80.6)
Male, n (%)	14 (19.4)
Age, mean (±SD) years	61.83 (10.30)
Total no. of G-CSF	
Filgrastim, n (%)	22 (26.8)
Pegfilgrastim, n (%)	52 (63.4)
Lenograstim, n (%)	5 (6.1)
Lipegfilgrastim, n (%)	3 (3.7)

SD = Standard deviation

**TABLE 3:** Baseline demographic characteristics of four types of g-CSF

	Filgrastim	Pegfilgrastim	Lenograstim	Lipegfilgrastim
Age, mean (±SD) years	65.05 ± 11.72	60.61 ± 9.66	55 ± 9.93	55 ± 15.01
Sex				
Female	16	42	4	3
Male	6	10	1	0
Time to onset (days ± SD)	11.73 ± 12.72	8.46 ± 4.16	6.67 ± 1.53	6.5 ± 3.10
Body temperature (°C)	38.55 ± 0.7807	38.67 ± 0.6865	39.8^a^	NA
CRP (mg/dL)	26.06 ± 15.39	24.81 ± 9.65	10.45 ± 9.914	NA

SD = standard deviation; CRP = C-reactive protein; NA, No detailed data available.

^a^ There is only one detailed data.

**FIGURE 3:**
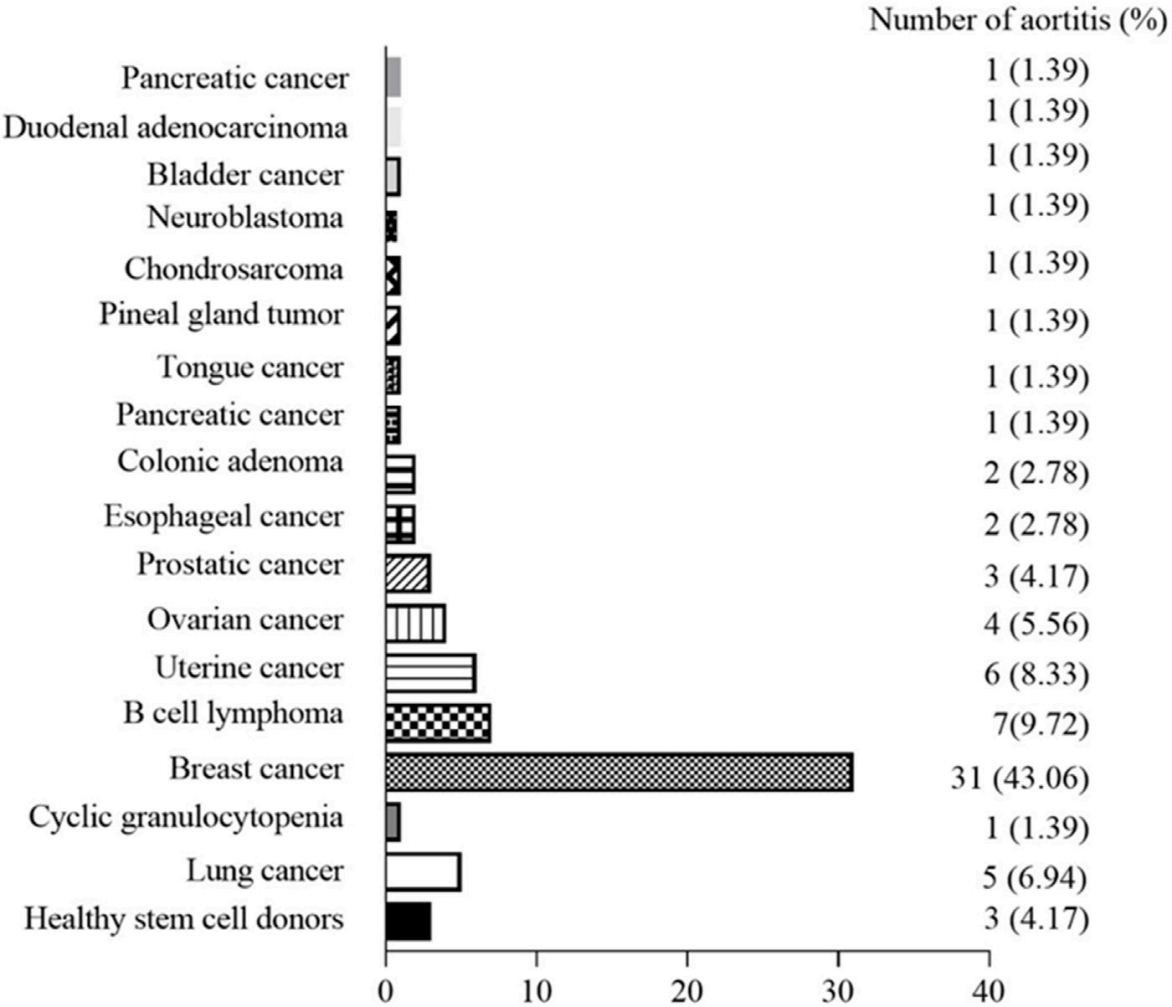
Graph shows cancer types among patients with G-CSF-induced aortitis.

**FIGURE 4:**
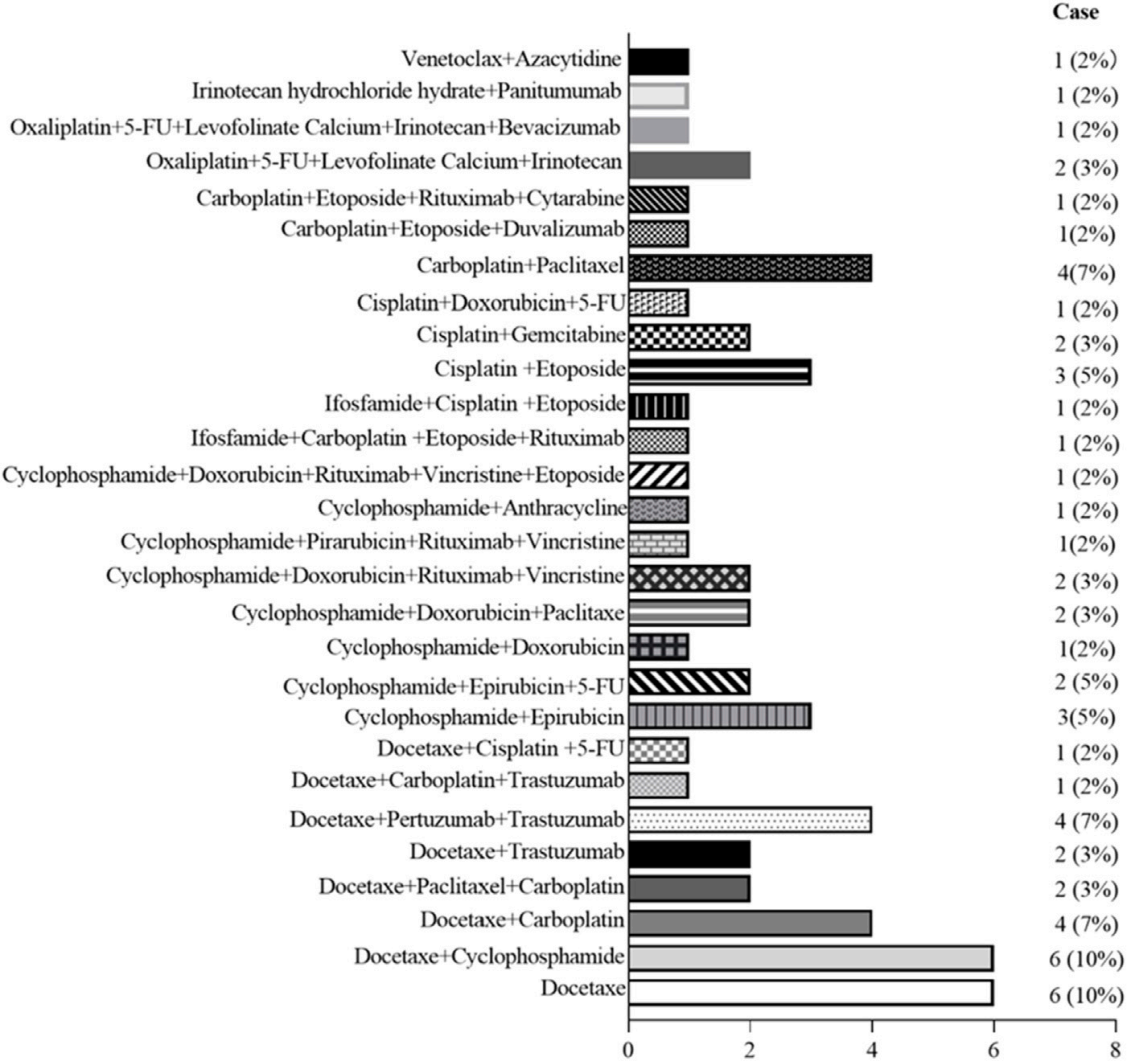
Graph shows the frequency of primary aortitis in patients with different chemotherapy regimen.

The relationship between the occurrence of G-CSF-induced aortitis and background diseases were shown in Figure 3. Apart from healthy stem cell donors (3 cases, 4.17%), G-CSF-induced aortitis was primarily found in patients with underlying malignancies (69 cases, 95.83%). Among them, the most common malignancies were breast cancer (31 cases, 43.06%), diffuse large B-cell lymphoma (7 cases, 9.72%) and uterine cancer (6 cases, 8.33%). The background chemotherapeutic agents for G-CSF-induced aortitis were shown in Table 4 and Figure 4. Common agents included docetaxel (26 cases), cyclophosphamide (14 cases), doxorubicin (10 cases) and carboplatin (10 cases), followed by cisplatin (7 cases), paclitaxel (7 cases), etoposide (7 cases) and trastuzumab (7 cases).

**TABLE 4:** Chemotherapeutic agents used in patients with g-CSF-induced aortitis.

Chemotherapy drugs	All
Docetaxel	26
Cyclophosphamide	14
Doxorubicin	10
Carboplatin	10
Cisplatin	8
Paclitaxel	7
Trastuzumab	7
Etoposide	7
5-fluorouracil	6
Rituximab	6
Epirubicin	5
Pertuzumab	4
Irinotecan	4
Vincristine	4
Oxaliplatin	3
Levofolinate Calcium	3
Gemcitabine	2
Duvalizumab	1
Panitumumab	1
Venetoclax	1
Azacytidine	1
Cytarabine	1
Bevacizumab	1

Note. Unless otherwise indicated, data are numbers of patients.

### 3.2 Clinical presentation and Radiologic findings of G-CSF-induced aortitis

**FIGURE 5:**
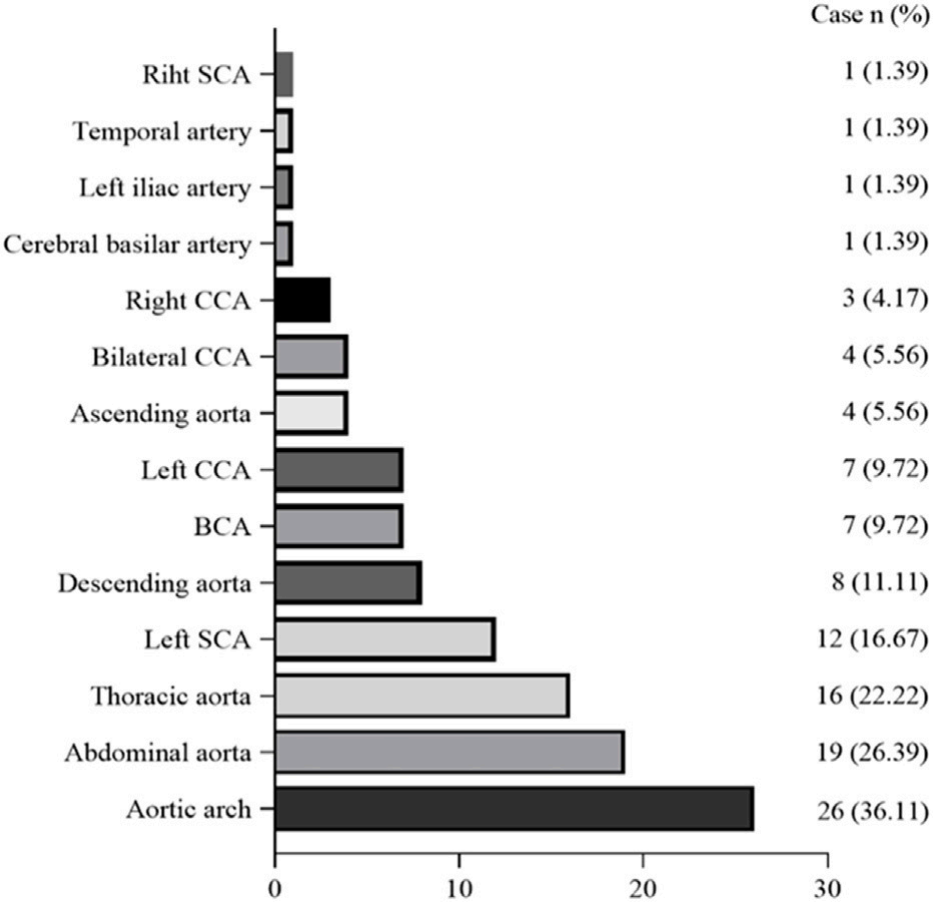
Graph shows distribution of G-CSF-induced aortitis at CT. Vertical axis indicates location of the aorta and its branches, and abscissa indicates the number of patients with G-CSF-induced aortitis. BCA = brachiocephalic artery; CCA = common carotid artery; SCA = subclavian artery.

The frequency of G-CSF-induced aortitis in patients with different chemotherapy regimens was shown in Figure 4. The chemotherapy regimen that was most frequently associated with the occurrence of arteritis was docetaxel and the combination of docetaxel and cyclophosphamide. The onset of aortitis may be presented as symptomatic or be asymptomatic. Symptomatic patients mainly presented with fever, chest pain, back pain, abdominal pain, neck pain, and sore throat, etc (Table 3). The blood test results revealed an elevated CRP (Table 3). The lesions were primarily located at the aortic arch in 26 cases (36.11%), abdominal aorta in 19 cases (26.39%) and thoracic aorta in 16 cases (22.22%) (Figure 5). The blood culture tests of all patients were negative. None of these patients met diagnostic criteria for conditions such as macro-arteritis, granuloma with polyangiitis, or giant cell arteritis. None of the patients had a history of IgG4-related diseases, and there were no other organ involvements attributed to IgG4-related diseases post the arteritis event.

Among the 72 patients with G-CSF-induced aortitis, 68 cases (96%) were symptomatic, while the remaining 4 (6%) were asymptomatic. Symptomatic patients with steroids or nonsteroidal anti-inflammatory drugs (NSAIDs) treatment exhibited an improvement in symptoms within an average of 15.26 ± 15.48 days, while untreated patients needed an average of 19.25 ± 10.23 days to achieve remission. Forty-seven patients accepted corticosteroids therapy, five were treated with NSAIDs, and the remaining patients did not receive any medication treatment, merely undergoing G-CSF discontinuation and conservative observation. For the 4 asymptomatic patients, after observation, the inflammatory changes in the aortitis also recovered. The specific recovery time of three asymptomatic patients with arteritis was unknown, and one asymptomatic patient recovered in 27 days. Five patients developed recurrent aortitis due to re-using the same type of G-CSF. Despite switching to another type of G-CSF, one patient still developed aortitis. CT findings indicated recovery in most cases, but one case led to aortic aneurysm, and two case led aortic dissection.

## 4 Discussion

Among all the published case reports, G-CSF-induced aortitis tended to affect elderly women over the age of 60. In addition, the cases of male G-CSF-induced aortitis observed in this review may reflect a higher likelihood of occurrence of prostatic cancer in men. The reason why G-CSF-induced aortitis is more likely to occur in females compared to males is not yet clear, so more samples would be needed to verify the gender differences in G-CSF-induced aortitis. Moreover, G-CSF-induced aortitis was frequently observed in patients with breast cancer as the primary tumor and in patients who received the anticancer drugs docetaxel and cyclophosphamide. The two drugs were used as combination therapy for patients with esophageal cancer (docetaxel and cyclophosphamide). Although, several reports have discussed the relationship between G-CSF-induced aortitis and anticancer drugs, especially docetaxel (Hoshina and Takei, 2021; Taimen et al., 2020). Our finding was that more patients with G-CSF induced aortitis have been using docetaxel, which is consistent with previous reports. However, the causal relationship between docetaxel and G-CSF-induced aortitis remains unclear. In addition, the drug instructions indicate that cyclophosphamide may lead to adverse reactions such as vasculitis. Therefore, when G-CSF is necessary, close attention should be paid to the clinical symptoms of patients when using cyclophosphamide in combination.

We discovered that over three-fourths of all cases occured in Asian populations, particularly in Japan and Korea. Human leucocyte antigen (HLA) as the genetic system with the richest polymorphism in humans, plays a crucial role in intercellular recognition, antigen recognition, and antigen presentation, resulting in different susceptibility of HLA genes to diseases. Evidence shows that HLA-DRB1*09:01 is one of the most common HLA-DRB1 alleles in Asians but is rare in European and American populations (Tsuchiya, 2013). Meanwhile, HLA-DRB1*09:01 has been shown to be associated with antineutrophil cytoplasmic antibody-associated vasculitis in Japan (Tsuchiya, 2013). In addition, previous literature has shown that the HLA-DRB1*04 and DRB1*07 alleles were strongly associated with aortitis in a Chinese Han population (Dang et al., 2002). The reason why G-CSF-induced aortitis was more common in Asian regions such as Japan and South Korea may be related to susceptibility genes in the Asian population that predispose to the development of aortitis. However, there is no evidence to support the correlation between HLA genes and G-CSF-induced aortitis. Therefore, it is necessary that further research will be conducted on the correlation between aortitis and HLA genes in populations prone to G-CSF- induced aortitis.

Our research findings also provided a deeper understanding of asymptomatic G-CSF-induced aortitis, which occurred in 4 out of 72 cases. These 4 asymptomatic patients with aortitis were found to have occurred between 1 and 2 weeks after using G-CSF through CT examination. Due to early detection, only G-CSF was discontinued for these asymptomatic patients and they did not receive treatment with glucocorticoids or NSAIDs. If G-CSF failed to discontinue medication timely in this type of patient, it was not clear whether it would lead to severe symptoms in the later stage. Therefore, considering the rarity of asymptomatic vasculitis and the issue of health economics, whether it is necessary to use ultrasound or CT for screening is not yet conclusive.

Currently, two types of G-CSF are clinically available, one being short-acting G-CSF including filgrastim and lenograstim, and another type being long-acting G-CSF including pegfilgrastim and lipegfilgrastim. We found that the proportion of pegfilgrastim was highest in G-CSF-induced aortitis cases, followed by filgrastim. In terms of pharmacokinetics, the half-life of filgrastim is 3.5 h, and that of pegfilgrastim is 33.2 h (Zamboni, 2003). Compared with filgrastim, pegfilgrastim was more likely to induce aortic inflammation, potentially due to its longer pharmacological effect. G-CSF-induced aortitis lesions were reported to be common in the aortic arch and proximal bifurcation. The distribution patterns of the aortitis are consistent with the findings of a single-center analysis by Takamatsu et al. (Lee et al., 2020). Owing to the unique anatomical structure of the aortic arch, hemodynamic instability heightens the risk of aortic dissection occurring at this location (Williams et al., 2022). Therefore, for patients with arteritis in the aortic arch and its branches, the treating physicians should be vigilant and actively treat to prevent the occurrence of aortic aneurysm and aortic dissection.

According to etiology, arteritis can be broadly classified as infectious and non-infectious (Benhuri et al., 2020). However, the underlying mechanism of aortitis-induced by G-CSF is still unclear. Functionally, G-CSF is a growth factor that regulates many aspects of neutrophil biology, including proliferation, differentiation, release, trafficking, and survival of granulocytes (Martin et al., 2021). However, in autoimmune and inflammatory diseases, the neutrophil response must be strictly regulated, as excessive recruitment and activation, or prolonged neutrophil survival time, can lead to chronic inflammation and sometimes irreversible organ damage (Martin et al., 2021). In this study, pegfilgrastim had the highest proportion of arterial inflammation, which may be related to its longer biological half-life.

G-CSF-induced aortitis exhibited good responsiveness to corticosteroids or NSAIDs and had a favorable prognosis. However, among all the collected cases, 2 cases had aortic dissection (Shiraki et al., 2022; Sato et al., 2017) and 1 case had aortic aneurysm (Miller et al., 2016). One case was a 58-year-old healthy male who was injected with filgrastim to donate bone marrow stem cells. Six months later, an iliac artery aneurysm was discovered (Miller et al., 2016). In the other two cases, after corticosteroids treatment, the symptoms of G-CSF-induced aortitis improved, but CT scans revealed aortic dissection (Shiraki et al., 2022; Sato et al., 2017). These cases suggested that even after the symptoms improve during corticosteroids therapy, there was still a risk of developing aortic dissection. Therefore, before corticosteroids reduction, it should be recommended to recheck CT to clarify the recovery status of arteritis.

There is controversy concerning the acceptability of re-administration or change dosage form of G-CSF in patients with a history of G-CSF-induced aortitis. Among all collected cases, three underwent pegfilgrastim re-administration later; three of the patients exhibited recurrence of pegfilgrastim-induced aortitis. One case who had clinically diagnosed pegfilgrastim-induced aortitis, after switching to filgrastim, still developed G-CSF-induced aortitis. One case who had clinically diagnosed filgrastim-induced aortitis, after switching to lenograstim, also reappeared with G-CSF-induced aortitis. This indicated that patients with a history of G-CSF-induced aortitis, whether through repeated use or changes in dosage form, cannot rule out the possibility of recurrent G-CSF-induced aortitis. However, considering the reporting bias, further validation of clinical data is needed for this possibility.

In conclusion, regardless of the dosage form of G-CSF, there is a risk of leading to arteritis. Due to the increased use of prophylactic treatment for chemotherapy-related neutropenia, the frequency of G-CSF-induced aortitis had also increased. In addition, G-CSF-induced aortitis also occured in healthy stem cell donors. Especially, asymptomatic patients with G-CSF-induced aortitis faced a greater risk of being missed by the attending physician. Given the regional characteristics of G-CSF-induced aortitis, it is recommended that physicians should pay close attention to the Asian population, especially elderly women after using G-CSF, to prevent the occurrence of complications of vasculitis.
